# Does Bariatric Surgery Reduce the Risk of Colorectal Cancer in Individuals with Morbid Obesity? A Systematic Review and Meta-Analysis

**DOI:** 10.3390/nu15020467

**Published:** 2023-01-16

**Authors:** Andrea Chierici, Paolo Amoretti, Céline Drai, Serena De Fatico, Jérôme Barriere, Luigi Schiavo, Antonio Iannelli

**Affiliations:** 1 Centre Hospitalier Universitaire de Nice—Digestive Surgery and Liver Transplantation Unit, Archet 2 Hospital, 151 Route Saint Antoine de Ginestière, BP 3079, CEDEX 3, 06200 Nice, France; 2 Faculté de Medicine, Université Côte d’Azur, 06000 Nice, France; 3 Medical Oncology Departement, Polyclinique Saint-Jean, 06800 Cagnes-sur-Mer, France; 4 Department of Medicine, Surgery and Dentistry, University of Salerno, 84081 Baronissi, Italy; 5 Inserm, U1065, Team 8 “Hepatic Complications of Obesity and Alcohol”, 06204 Nice, France

**Keywords:** bariatric surgery, colorectal cancer, mortality, risk

## Abstract

Bariatric surgery has shown to be effective in producing sustained weight loss and the resolution of obesity related medical problems. Recent research focused on the role of obesity and adipose tissue in tumorigenesis, finding a strong crosslink through different mechanisms and highlighting an increase in cancer incidence in individuals with obesity. The aim of this meta-analysis is to find if bariatric surgery reduces the incidence of colorectal cancer in patients with obesity. We performed a meta-analysis including 18 studies (PROSPERO ID: CRD4202235931). Bariatric surgery was found to be significantly protective toward colorectal cancer incidence in individuals with obesity (HR: 0.81, *p* = 0.0142). The protective effect persisted when considering women (RR: 0.54, *p* = 0.0014) and men (RR: 0.74, *p* = 0.2798) separately, although this was not significant for the latter. No difference was found when comparing Roux-en-Y gastric bypass and sleeve gastrectomy. Bariatric surgery reduces the incidence of colorectal cancer in individuals with obesity independently from gender and surgical procedure. Prospective large cohort studies are needed to confirm these findings.

## 1. Introduction

Colorectal cancer (CRC) is the world’s third most frequent cancer and the second major source of cancer-related deaths, with an estimated 1.8 million new cases and 881,000 deaths in 2018 [1], and obesity has been shown to be associated with an increased risk of CRC [2]. According to recent statistics, the number of individuals with obesity or overweight has surpassed 2 billion—approximately 30% of the world’s population [3]. Epidemiological data suggest that obesity is associated with a 30–70% increased risk of CRC in men, whereas the association is less consistent in women [4]. Renehan et al. [5] showed that the obesity-related risk for CRC increases by 24% in men and 9% in women per 5 kg/m^2^ increase in BMI. With sustained long-term weight loss and remission or improvement of comorbidities associated with obesity, bariatric surgery (BS) is regarded as the most effective treatment for morbid obesity and its associated medical problems [6]. These findings naturally lead to a question: do patients with a history of BS have a lower risk of developing CRC than their non-operated on counterparts? The purpose of this meta-analysis is to investigate the impact of BS on the risk of developing CRC in individuals with obesity. Previous meta-analyses have reported that the risk of cancer is reduced after BS [7,8,9], but they included studies which are highly heterogeneous in terms of follow-up without conducting any specific analysis to overcome this bias. Moreover, several large cohort studies on a possible protective effect of BS against CRC and cancer in general have been published more recently. In light of the aforementioned criticism, our aim is to produce an updated meta-analysis elucidating the role of BS in CRC incidence.

## 2. Materials and Methods

### 2.1. Search Strategy

We performed a systematic review of the literature following the Preferred Reporting Items for Systematic Reviews and Meta-Analyses (PRISMA) and AMSTAR (Assessing the methodological quality of systematic reviews) guidelines [10,11], and the MOOSE (Meta-analysis of Observational Studies in Epidemiology) recommendations.

We used the PROSPERO platform to register the protocol of this research (ID: CRD4202235931). The PubMed, Scopus, Embase, and Cochrane Library databases were screened without time restrictions up to 31 August 2022.

Full texts were obtained through the University of Milan (Università degli Studi di Milano) digital library and the University of Nice (Université Côte d’Azur) digital library, or by direct contact with the authors. In the search strategy we also included studies and previous reviews on the topic, and their references were screened to find additional relevant studies according to selection criteria. 

### 2.2. Selection Criteria

Following PRISMA recommendations, we realized a specific population (P), intervention (I), comparator (C), outcome (O), and study design (S) (PICOS) framework to define study eligibility:

Population (P): adult individuals (≥18 years old), diagnosed with morbid obesity, followed-up for at least 3 years to investigate the insurgence of CRC;

Intervention (I): BS;

Comparison (C): simple observation or any behavioral or medical treatment;

Outcomes (O): risk to develop a CRC during the follow-up period;

Study design (S): retrospective and prospective comparative studies with at least 10 patients per group.

### 2.3. Exclusion Criteria

All non-comparative studies describing the risk to develop CRC in populations of individuals with morbid obesity undergoing BS or not were not eligible for inclusion. Studies not reporting a specific risk measure to develop CRC were also excluded. Only studies with the full text available and English-language research were considered for analysis.

### 2.4. Systematic Review Process

In the first step, 1407 articles were identified by the literature search. Duplicates found through different databases were removed using Mendeley reference software (Mendeley Ltd., London, UK). A screening of the titles and abstracts of 723 records was then performed, based on an a screening form that was created to guide study selection. Investigators (PA, AC, CD, SDF) were blinded to each other’s decisions. 

### 2.5. Risk of Bias Assessment 

The risk of bias was assessed for the selected studies according to the ROBINS-I tool as recommended by the Cochrane Collaboration [12]. This tool explores overall and specific risk of bias defining different bias domains: (1) bias due to confounding; (2) bias in the selection of participants into the study; (3) bias in classification of interventions; (4) bias due to deviations from the intended interventions; (5) bias due to missing data; (6) bias in the measurement of the outcomes; (7) and bias in the selection of the reported results. 

Investigators (JB, LS) collected risk of bias data according to the methodology proposed by Higgins [13]. Finally, we created bar and traffic light plots to graphically display the results of the risk of bias assessment.

### 2.6. Data Extraction and Assessment of Included Studies

A computerized spreadsheet (Microsoft Excel 2021; Microsoft Corporation, Redmond, WA, USA) was developed to collect information about study design and methodology, participant demographics and baseline characteristics, obesity treatment details, risk of colorectal cancer insurgence, and survival.

### 2.7. Primary and Secondary Endpoints

Primary outcome was represented by identifying the relative risk (RR) of developing CRC in a population of morbidly obese patients who underwent BS compared to morbidly obese patients who did not have BS.

In the same subset, secondary outcomes consisted in defining the RR of colonic and rectal cancer separately. Moreover, a subgroup analysis considering CRC RR for men and women separately and depending on the type of BS performed (SG vs. RYGB) were realized. Finally, we identified the hazard ratio (HR) of colorectal cancer in individuals with obesity undergoing BS or not.

### 2.8. Statistical Analysis

We performed a meta-analysis of binary outcomes and hazard ratios, so primary and secondary outcome measures were expressed in terms of RR or HR and 95% Confidence Intervals (CI) for cancer risk.

Fixed and random effects models based on the Mantel-Haenszel method were built displaying the impact of heterogeneity on the results. When low heterogeneity (<25%) was found, a fixed-effects model was chosen to compute the outcome. The eventual presence of outliers was investigated; if any were found, its effect size was excluded.

Heterogeneity between studies was quantified by I^2^ statistic and Cochran’s Q test; cut-off values of 25%, 50%, and 75% were considered as low, moderate, and high, respectively [14]. Sensitivity analyses were conducted after inspecting the patterns of effect sizes and the heterogeneity of the included studies. This was done through Graphic Display of Heterogeneity (GOSH) plots; if we found any study predominantly responsible for heterogeneity, we conducted a sensitivity analysis excluding them.

To explore publication bias we developed funnel plots. Egger’s test of the intercept was then used to quantify the funnel plots’ asymmetry when feasible. 

A statistical analysis was conducted with RStudio software (RStudio: Integrated Development Environment for R—ver. 2022.07.02) [15], using the “meta”, “metafor”, “robvis”, and “dmetar” packages [16,17,18,19].

## 3. Results

**Figure 1:**
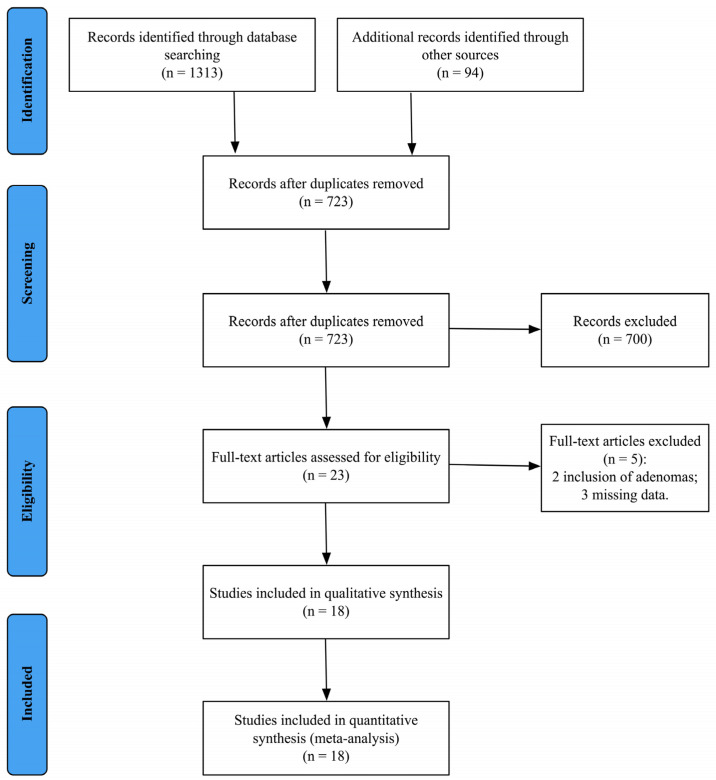
PRISMA flow-chart depicting the overall review process.

As shown in Figure 1, we included 18 [2,6,20,21,22,23,24,25,26,27,28,29,30,31,32,33,34,35] studies in the quantitative and qualitative analysis. A meta-analysis was conducted on 12,517,893 patients. Characteristics of the included studies are reported in Table 1.

### 3.1. Colorectal Cancer

Data to evaluate the primary outcome was retrieved from 17 studies [2,6,20,21,22,23,25,26,27,28,29,30,31,32,33,34,35].

**Figure 2:**
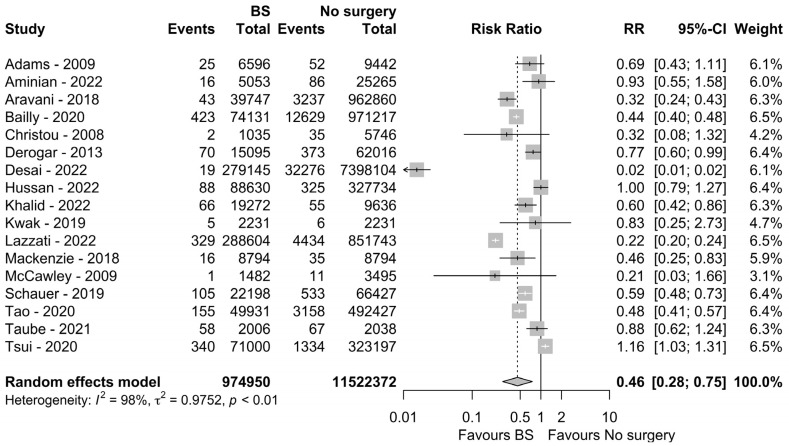
Forest plot comparing colorectal cancer incidence in individuals with obesity who did or did not undergo BS [2,6,20,21,22,23,25,26,27,28,29,30,31,32,33,34,35].

Individuals with morbid obesity who underwent BS had a 54% reduction in the risk of developing CRC during follow-up (RR: 0.46, 95% CI: 0.28–0.75, *p* = 0.018, I^2^ = 98%). A forest plot is shown in Figure 2. 

A sensitivity analysis was conducted due to high heterogeneity. Four studies [26,27,30,35] were found to be mainly responsible for the heterogeneity, but their exclusion failed to reduce it consistently; an advantage for individuals with obesity and a history of BS was still highlighted (RR: 0.57, 95% CI: 0.47–0.69, *p* = 0.0001, I^2^ = 75%).

Only three studies reported data concerning colonic and rectal cancer separately [2,27,34]. 

For colonic cancer, a meta-analysis showed a tendency in favor of BS patients, although this was not significant (RR: 0.75, 95% CI: 0.46–1.21, *p* = 0.2444, I^2^ = 89%).

Similarly, considering rectal cancer, a non-significant tendency in the advantage of BS patients was found (RR: 0.74, 95% CI: 0.4–1.39, *p* = 0.3523, I^2^ = 87%).

### 3.2. Subgroup Analysis

From literature research we identified five studies [6,22,25,27,34] reporting the incidence of CRC only for men in the BS and in the non-surgical groups. A metanalysis showed a protective effect tendency against CRC for men with a history of BS, but this was not significant (RR: 0.74, 95% CI: 0.43–1.28, *p* = 0.2798, I^2^ = 96%).

We found a significant (46%) reduction of risk of CRC (RR: 0.54, 95% CI: 0.37–0.79, *p* = 0.0014, I^2^ = 90%) in women with a history of BS. Six studies [6,22,25,27,32,34] reported specific cancer incidence data for women.

Considering the type of bariatric procedure, SG and RYGB were the most common. A binary outcome meta-analysis on five studies [6,22,24,31,35] showed that there was no difference in the risk of developing CRC irrespective of whether the patients had a history of SG or an RYGB (RR: 1.03, 95% CI: 0.72–1.47, *p* = 0.8713, I^2^ = 45%). Only three [6,31,35] studies reported data after laparoscopic adjustable gastric banding, so this technique was not included in the meta-analysis. 

### 3.3. Meta-Analysis of HR

**Figure 3:**
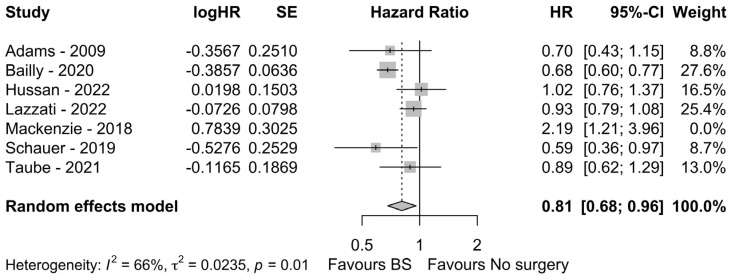
Forest plot of HR meta-analysis after sensitivity analysis [6,20,27,30,31,33,34].

Finally, we selected those articles reporting HR estimates of developing CRC in individuals with obesity whether they had a history of BS or not [6,20,27,30,31,33,34]. The meta-analysis of HR failed to find a significant HR estimate in favor of the BS or the non-surgical group, although a tendency toward the BS group was found (HR: 0.88, 95% CI: 0.69–1.12, *p* = 0.2974, I^2^ = 77%). However, after sensitivity analysis and exclusion of the research by Mackenzie et al. [31], which was identified as the main contributor to heterogeneity, a significant 19% reduction of HR for those obese patients who had a history of BS was found (HR: 0.81, 95% CI: 0.69–0.96, *p* = 0.0142, I^2^ = 66%), as shown in Figure 3.

### 3.4. Assessment of Publication Bias and Risk of Bias

**Figure 4:**
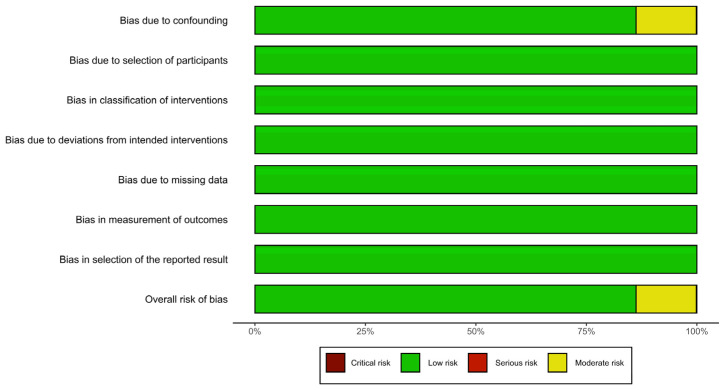
Risk of bias barplot with details of risk of bias for each bias domain.

Most of the included studies resulted in having a low risk of bias after assessment through the ROBINS-I tool. A moderate risk of bias was identified in nine [2,20,21,22,23,24,25,32,34] studies, always related to bias due to confounding depending on differences in the baseline characteristics of the study groups when the authors did not apply statistical strategies to reduce between group disparities (Figure 4 and Figure S1).

To assess publication bias, we generated contour-enhanced funnel plots for the primary outcome (Figure S2). The Egger’s test of the intercept did not indicate the presence of funnel plot asymmetry (int: −0.069, 95% CI: −5.54–5.4, *p* = 0.9807).

## 4. Discussion

The tumorigenic effect of obesity has been widely investigated in the last decades and particularly in the last years, along with the recent explosion of the “obesity epidemic”. As BS has been shown to be associated with significant and durable weight loss and remission of obesity-linked comorbid conditions, the possibility that BS could be effective in reducing the risk of cancer has gained increasing interest. Currently, it is accepted that obesity is a risk factor for several cancers including endometrial, ovarian, female breast, colorectal, pancreatic, esophagus, and kidney cancer [36]. Many large cohort studies comparing cancer incidence in individuals with obesity with or without a history of BS showed an important protective effect of BS for many types of cancers. For example, Lazzati et al. [30] recently conducted an analysis with inverse probability treatment weighting and a propensity score matching approach on 1,140,347 patients from the national French discharge database (PMSI), with the aim of comparing the incidence of different cancers in two cohorts of individuals with morbid obesity with and without a history of BS. The authors showed a protective effect of BS on obesity-related cancers (HR: 0.89), critically reducing the effects of confounders respecting the treatment exchangeability assumption. As expected, for non-obesity-related cancers, BS did not result in having a protective effect. Through the same rigorous statistical method, Rustgi et al. [37] recently analyzed the incidence of cancers in 98,090 severely obese (BMI > 40) patients with non-alcoholic fatty liver disease selected from a population of 2,043,936 individuals with obesity. The 33,435 patients who underwent BS showed a reduction in the risk of developing any type of cancer (HR: 0.82), which was even more accentuated when considering only obesity-related cancers (HR: 0.65).

On the other hand, recent literature has shown a possible relationship between the anatomical gut modifications due to BS and an increased risk of colorectal cancer [38]. As a matter of fact, certain bariatric procedures, such as RYGB, are based on anatomical bowel rearrangements responsible for the gut microbiota and a colonic exposure to an elevated concentration of bile acids. These microbiota alterations basically consist in an overgrowth of pro-inflammatory flora and a reduction in butyrate-producing bacteria. The latter is a short chain fatty acid with well-known anti-inflammatory and anti-carcinogenic properties. However, few animal and human studies have investigated the real effects of this surgery on colorectal cancer incidence and the above-mentioned mechanisms still remain hypothetical. 

In the present study, we aimed to focus only on CRC incidence to also explore possible subsets additionally favoring cancer incidence. As mentioned earlier, meta-analyses have been previously performed on this topic. Firstly, Almazeedi et al. [8] reviewed seven studies involving 1,213,727 patients after various bariatric operations (SG, RYGB, gastric banding and duodenal switch); the authors reported that the risk of CRC decreased by 35% in individuals with a history of BS. These data were substantially confirmed by Janik et al., who analyzed 13 studies and a total number of 3,233,044 patients, finding a 37% reduction in the risk of developing CRC compared to individuals with obesity with no previous history of BS [9].

In our meta-analysis we included 18 studies involving more than 12 million patients, and we found that individuals with morbid obesity who underwent BS had a 54% reduction in the risk of developing CRC during follow-up. However, this effect must be analyzed, taking into account the heterogeneity of the study characteristics, the populations, and the results of the studies included in the meta-analysis. 

Firstly, although most of the studies on the topic found a protective effect of BS, others came to opposite conclusions. Derogar et al. [25] indicated that BS is associated with an increased risk of CRC with increasing time after BS. The study reports a standardized incidence ratio, which is the observed number of cases in a cohort divided by the expected number of cases for that group. The expected number was calculated by multiplying a person’s exposure time by age-, sex- and calendar-year-specific incidence of a specific condition for the general “normal-risk” population. The use of the SIR gives a robust measure of relative risk for the comparison of cohorts when the cohorts are not sufficiently well matched to allow a direct case–control comparison. However, when calculating the RR of developing CRC for BS patients, it resulted in a significative reduction in risk (RR: 0.77, 95% CI: 0.60, 0.99). Mackenzie et al. [31] also found contradictory results, as they reported that patients undergoing BS have a more than two-fold risk (odds ratio: 2.19) of developing CRC compared to individuals with obesity with no history of BS. However, again, an important bias due to confounders was found in population comparisons, as data were retrieved from the Hospital Episode Statistics, in which obesity was coded only as a co-morbidity, and exact weight and BMI data were not available. On the other hand, we cannot ignore how the studies by Adams et al. [20], Aminian et al. [21], Aravani et al. [22], Christou et al. [23], Ciccioriccio et al. [24], McCawley et al. [32], Tao et al. [2], and Taube et al. [34] were also shown to have a moderate risk of bias due to confounding, as shown in Figure S1.

Second, when focusing on the characteristics of follow-up, an important between-studies heterogeneity is noticed, with follow-up time varying from 3 to more than 20 years, strongly influencing the results in a strictly “time-dependent” condition like cancer. As a matter of fact, cancer incidence increases when follow-up is longer, and it is obvious that studies with a short follow-up have limited reliability compared to those with very long follow-up. Previous meta-analyses on this subject compare the incidence of colorectal cancer in populations with very different follow-ups and, consequently, very different chances to identify colorectal cancer. In these studies, odds ratios (OR) and risk ratios (RR), which are strictly time dependent, were used to measure the risk of colorectal cancer. This represents the most consistent limitation, as studies reporting risk estimates depending on time cannot be effectively compared when follow-up time is different, especially in cases of highly dissimilar time spans. An important novelty introduced by this meta-analysis concerns how we succeeded in overcoming this issue. The risk of colorectal cancer in the two populations (individuals with obesity who underwent bariatric surgery vs individuals with obesity who did not undergo bariatric surgery) have been estimated through a risk measure which is independent of time. Our meta-analysis is based on hazard ratio (HR) as a measure of colorectal cancer risk. HR is time independent and permits the identification of the risk of developing colorectal cancer at the moment of bariatric surgery, independently of follow-up time. This makes populations with different follow-ups directly comparable, with no methodological bias. We selected those articles reporting HR estimates of developing CRC in individuals with obesity, whether they underwent BS or not [12,14,17,18,19,20,21] finding a significant 19% reduction of HR associated with a previous history of BS.

A gender-based subgroup analysis also found a protective role played by BS, as expected from the main outcome. The fact that no risk difference was found between patients who underwent SG and those who had RYGB confirms that the reduction of the risk of CRC mainly depends on the reduction of body weight, which translates to the reduction of the amount of adipose tissue and its related tumorigenic effect and not on a procedure-specific mechanism. As a matter of fact, recent research has shown how white adipose tissue is responsible for initiation and progress through different processes in individuals with obesity [39]. First of all, obesity induces an alteration in immune cell homeostasis maintenance, promoting systemic inflammation, growth, tumor angiogenesis, and the production of reactive oxygen species. Secondly, obesity leads to increased DNA damage and reduced DNA repair. Moreover, an abnormal increase in white adipose tissue seems to favor mutations in adipose stroma cells, which become mobilized, and then migrate and play an important role in tumor progression. Fat tissue also represents the favorite source of energy of carcinomas and the increased β-oxidation of lipids is a marker of tumor aggressiveness. Although Hussan et al. [27] stated that RYGB and not SG is related to a reduction in CRC incidence, this could simply depend on an increased white adipose tissue loss in those patients who underwent RYGB, as also stated by the authors themselves. Thus, the actual shift from RYGB to SG as the most performed BS worldwide [40] should not influence the benefits of BS on CRC incidence.

Another important novelty introduced by our meta-analysis compared to the previous ones is that in the last 2 years, a number of large new cohort studies, which were not included even in the last meta-analysis, have been published on this subject. As a matter of fact, our meta-analysis has a four-fold increased population (12,000,000) compared to the previous one. Moreover, these recent cohort studies are based on very different populations, amplifying the applicability of our results. In addition, these same studies, compared to the ones included in previous meta-analyses, have contrasting results, not always in accordance with a protective effect of bariatric surgery, which made it interesting to realize a new analysis.

Although subset analyses were performed to overcome biases, our study still have some limitations depending on the above-mentioned factors. One important source of between study heterogeneity is represented by the different inclusion criteria used to create the study cohorts. For example, different BMI cut-offs (35 kg/m^2^ or 40 kg/m^2^) were set, different patients’ characteristics were analyzed to compare populations, and different bariatric procedures were realized; the study by McCawley et al. [32] only included women. Moreover, all the included studies are retrospective and the modality to retrieve data during the follow-up was again quite heterogenous and not always precisely specified in the methods.

## 5. Conclusions

BS has proven to be effective in inducing weight loss, obesity-related comorbidity remission and also in reducing cancer incidence. This meta-analysis confirms the protective role of BS against CRC through both RR and HR evaluation, which is independent of gender and the type of surgical procedure performed. Further studies are needed to confirm these results.

## Figures and Tables

**Table 1:** Detailed characteristics of the included studies [2,6,20,21,22,23,24,25,26,27,28,29,30,31,32,33,34,35].

Author	Year	Country	BS *		Control *		Follow-Up (y)	Risk Estimate ^†^ (95% CI)
			Total	Events	Total	Events		
Adams et al.	2008	USA	6596	25 (0.004)	9442	52 (0.006)	12.3	HR: 0.7 (0.43–1.15)
Aminian et al.	2022	USA	5053	16 (0.003)	25,265	86 (0.003)	6.1	-
Aravani et al.	2018	UK	39,747	43 (0.001)	962,860	3237 (0.003)	3	-
Bailly et al.	2020	FR	74,131	423 (0.006)	971,217	12629 (0.013)	5.7	HR: 0.68 (0.6–0.77)
Christou et al.	2008	CA	1035	2 (0.002)	5746	35 (0.006)	5	RR: 0.32 (0.076–1.313)
Ciccioriccio et al.	2021	IT	20,571	22 (0.001)	-	-	4.3	-
Derogar et al.	2013	SE	15,095	70 (0.005)	62,016	373 (0.006)	10	-
Desai et al.	2022	USA	279,145	19 (0.0001)	7,398,104	32,276 (0.004)	-	OR: 0.06 (0.04–0.1)
Hussan et al.	2022	USA	88,630	88 (0.001)	327,734	325 (0.001)	3	HR: 1.02 (0.76–1.37)
Khalid et al.	2019	USA	19,272	66 (0.003)	9636	55 (0.006)	5	-
Kwak et al.	2019	USA	2231	5 (0.002)	2231	6 (0.002)	7.8	OR: 0.62 (0.42–0.91) ^§^
Lazzati et al.	2022	FR	288,604	329 (0.001)	851,743	4434 (0.005)	5.7	HR: 0.93 (0.79–1.08)
Mackenzie et al.	2018	SE/UK	8794	16 (0.002)	8794	35 (0.004)	4.6	HR: 2.19 (1.21–3.96)
McCawley et al.	2009	USA	1482	1 (0.0007)	3495	11 (0.003)	-	-
Schauer et al.	2019	USA	22,198	105 (0.005)	66,427	533 (0.008)	3.5	HR: 0.59 (0.36–0.97)
Tao et al.	2019	DK/SE/NO/FI/IS	49,931	155 (0.003)	492,427	3158 (0.006)	3.1	-
Taube et al.	2021	SE	2006	58 (0.03)	2038	67 (0.03)	22.2	HR: 0.89 (0.62–1.28)
Tsui et al.	2020	USA	71,000	340 (0.005)	323,197	1334 (0.004)	-	-
			995,521		11,522,372			

* Crude incidence data refers to colorectal cancer through the entire follow-up, and it is expressed as n (%). ^†^ Risk estimates refers to the risk of developing colorectal cancer in obese patients who underwent BS compared to those who did not. HR: Hazard Ratio; OR: Odds Ratio. ^§^ In this case OR refers to the risk to develop colorectal lesions, including benign lesions.

## Data Availability

The data is available through a specific request to the authors.

## Supplementary Materials

The following supporting information can be downloaded at: https://www.mdpi.com/article/10.3390/nu15020467/s1, Figure S1: traffic light plot showing risk of bias assessment for each specific domain for each study; Figure S2: funnel plot exploring publication bias and the result of the relative Egger’s test of the intercept.
